# Complete Radiotherapy Response of Chemoresistant Para-Aortic Lymph Node Metastases in an Endometrial Cancer Patient Meeting the Amsterdam II Criteria for Lynch Syndrome

**DOI:** 10.7759/cureus.107546

**Published:** 2026-04-22

**Authors:** Isao Otsuka, Kazufusa Shoji

**Affiliations:** 1 Obstetrics and Gynecology, Kameda Medical Center, Kamogawa, JPN; 2 Radiology, Kameda Medical Center, Kamogawa, JPN

**Keywords:** amsterdam ii criteria, chemoresistance, endometrial cancer, lymph node metastasis, lynch syndrome, radiation therapy

## Abstract

Patients with Lynch syndrome often have a strong family history of colorectal and endometrial cancers and exhibit mismatch repair (MMR) deficiency, which may be associated with chemoresistance. Radiotherapy can enhance tumor-specific immune responses and may be effective in such patients. A 49-year-old perimenopausal woman with a family history of colon cancer in three relatives was diagnosed with endometrial carcinoma with para-aortic lymph node metastases. She underwent surgical staging, including pelvic and para-aortic lymphadenectomy. Pathological examination revealed a mixed endometrioid (grade 2) and serous carcinoma. Lymph node metastases were identified in 15 of the 25 nodes removed (including 7 of 10 para-aortic nodes). Although she received adjuvant taxane/platinum-based chemotherapy, para-aortic nodal recurrence developed three months after completion of treatment. Radiotherapy was administered, resulting in complete regression of the para-aortic nodes. She remains well with no evidence of disease 150 months after surgery. The patient meets the Amsterdam II criteria for Lynch syndrome but without molecular confirmation. MMR-deficient tumor cells contain numerous frameshift peptides resulting from DNA mismatch, and tumors exhibit significant infiltration of tumor-infiltrating lymphocytes. Ionizing radiation induces tumor cell death and neoantigen release, resulting in the trafficking of T cells to the tumor, which, hypothetically, may convert the irradiated tumor into an efficient, individualized in situ vaccine. Radiotherapy may be a promising option for chemoresistant lesions in selected, similar cases. Further studies are warranted to identify the specific subgroup of patients who are likely to benefit from radiotherapy.

## Introduction

Lynch syndrome is an autosomal dominant inherited disorder caused by germline pathogenic variants in the mismatch repair (MMR) genes, i.e., *MLH1, MSH2, MSH6*, and *PMS2*, as well as *EPCAM*. Diagnosis is confirmed by the identification of these germline variants by DNA sequencing. These variants are associated with increased risks of colorectal and endometrial cancers, as well as other cancer types. The Amsterdam II criteria, based on clinical information alone, have a positive predictive value of 57% for its diagnosis [1].

MMR deficiency is a hallmark of Lynch syndrome-associated cancers. While immune checkpoint inhibitors are effective in these tumors by activating tumor-specific effector T cells, MMR deficiency may be associated with a poor response to platinum-based chemotherapy in both metastatic endometrial [2-4] and colorectal cancers [5]. Since radiation can enhance tumor-specific immune responses by increasing T-cell priming [6], radiotherapy may be effective for MMR-deficient tumors [3,7-9]. Herein, we report a case of chemoresistant para-aortic lymph node metastases from endometrial carcinoma (progressing within six months of platinum/taxane chemotherapy) that showed a complete durable response to radiotherapy in a patient meeting the Amsterdam II criteria for Lynch syndrome.

## Case presentation

A 49-year-old perimenopausal woman, gravida 2, para 2, presented to Kameda Medical Center in Kamogawa, Japan, with irregular genital bleeding. She had a family history of colon cancer in her mother, maternal grandfather, and maternal aunt, diagnosed at ages 57, 80, and 60 years, respectively. She had a body mass index of 20.0 kg/m² and an ECOG performance status of 0. Two months before surgery, she began treatment with prednisolone for membranous nephropathy.

**Figure 1:**
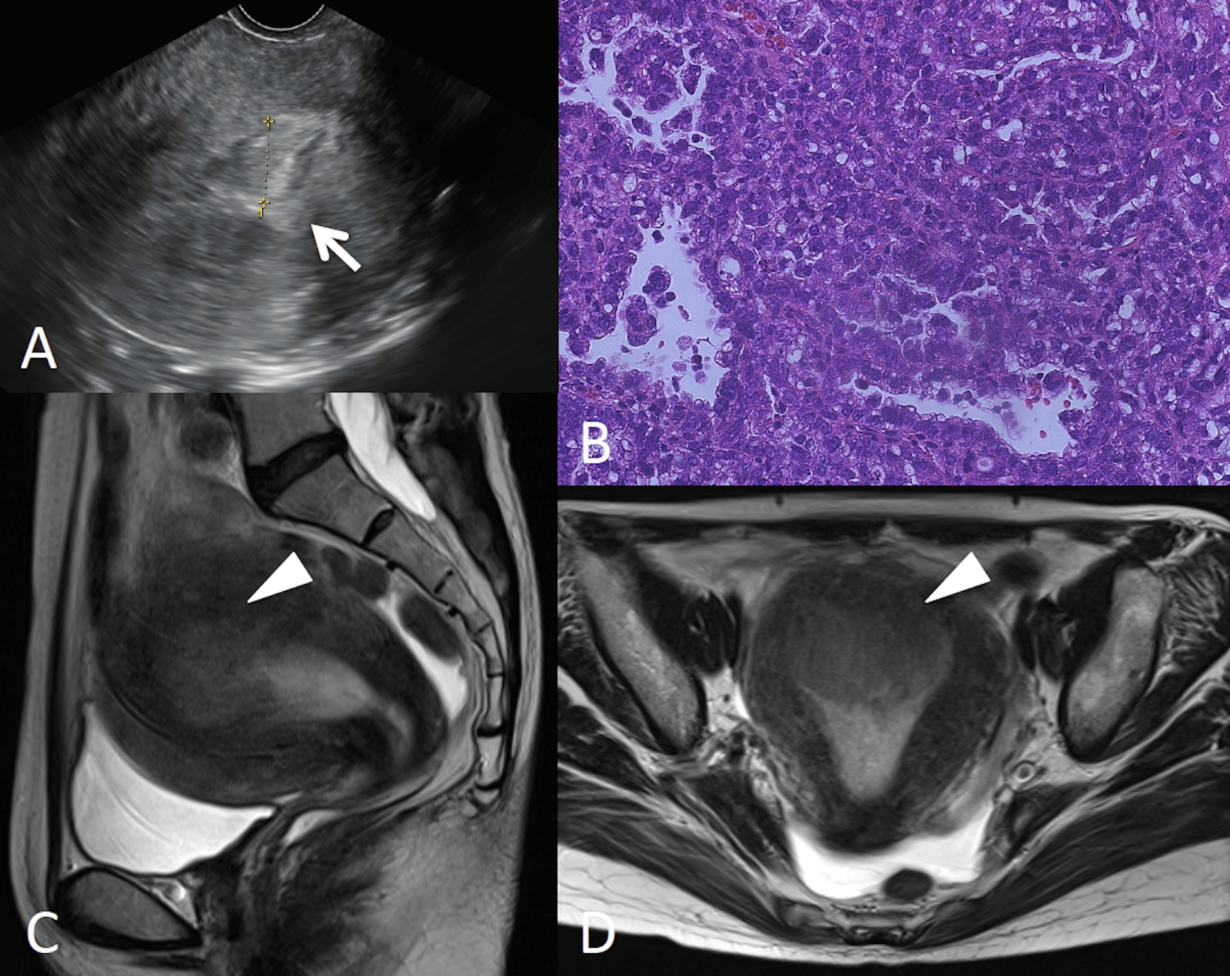
Vaginal ultrasonography shows endometrial thickening (arrow) (A). Endometrial sampling demonstrates a grade 2 endometrioid carcinoma (B). T2-weighted magnetic resonance image of the pelvis reveals a 6‑cm intrauterine tumor (C, sagittal section; D, axial section; arrowhead) without pelvic lymph node enlargement.

Vaginal ultrasonography showed endometrial thickening (Figure 1A), and endometrial sampling demonstrated a grade 2 endometrioid carcinoma (Figure 1B). The serum CA‑125 level was 138 U/mL. Pelvic magnetic resonance imaging revealed a 6‑cm intrauterine tumor without pelvic lymph node enlargement (Figures 1C, 1D).

**Figure 2:**
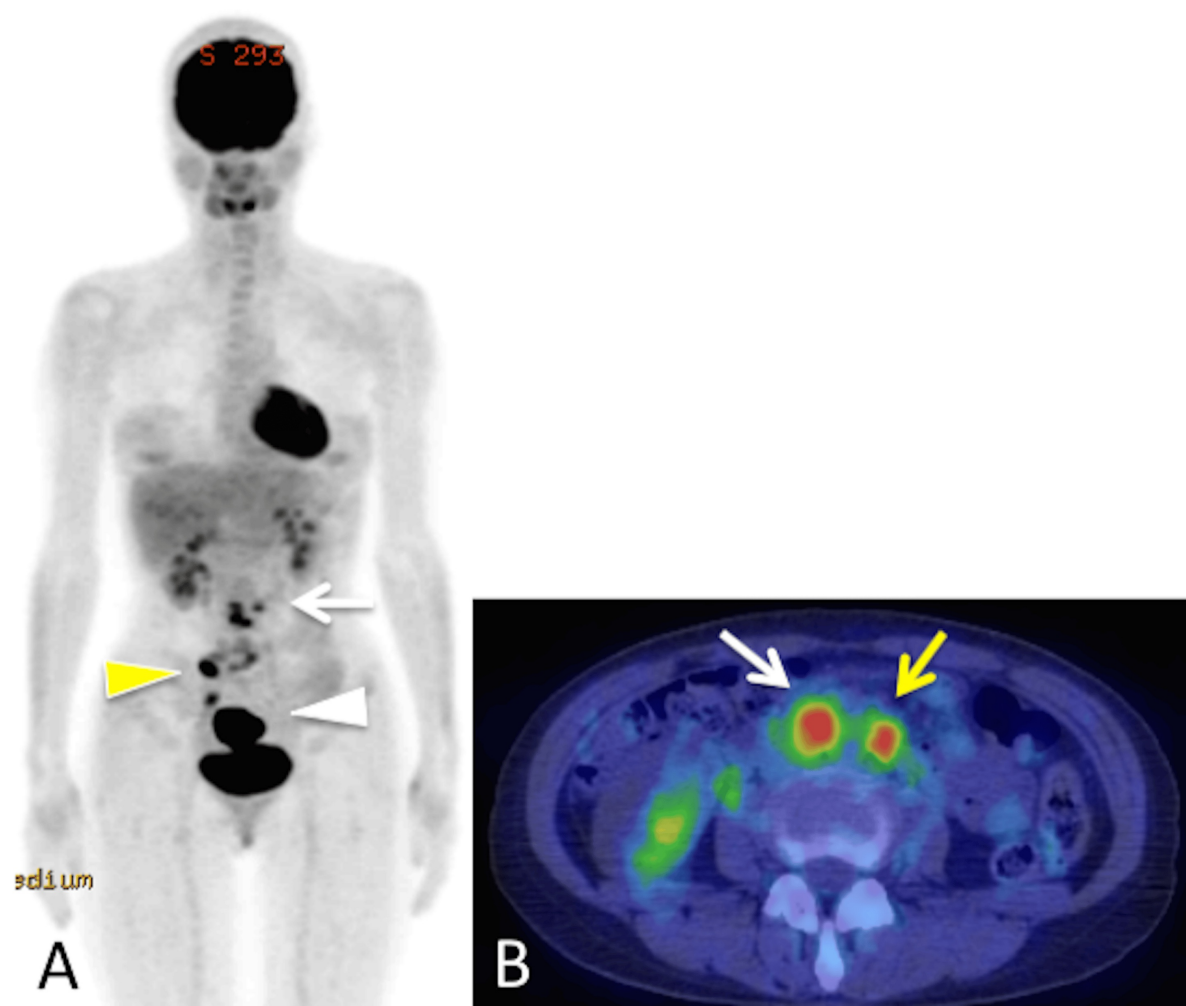
18F-fluorodeoxyglucose (FDG) PET/CT demonstrates abnormal uptake in para-aortic (maximum standardized uptake value (SUVmax), 12.08) (white arrow) and right pelvic lymph nodes (yellow arrowhead) in addition to the uterine cavity (SUVmax, 18.03) (white arrowhead) (A). FDG uptake in para-aortic lymph nodes: aortocaval (white arrow) and left para-aortic nodes (yellow arrow) (B).

^18^F-fluorodeoxyglucose PET/CT demonstrated abnormal uptake in para-aortic lymph nodes (maximum standardized uptake value (SUV_max_), 12.08) in addition to the uterine cavity (SUV_max_, 18.03) (Figure 2).

**Figure 3:**
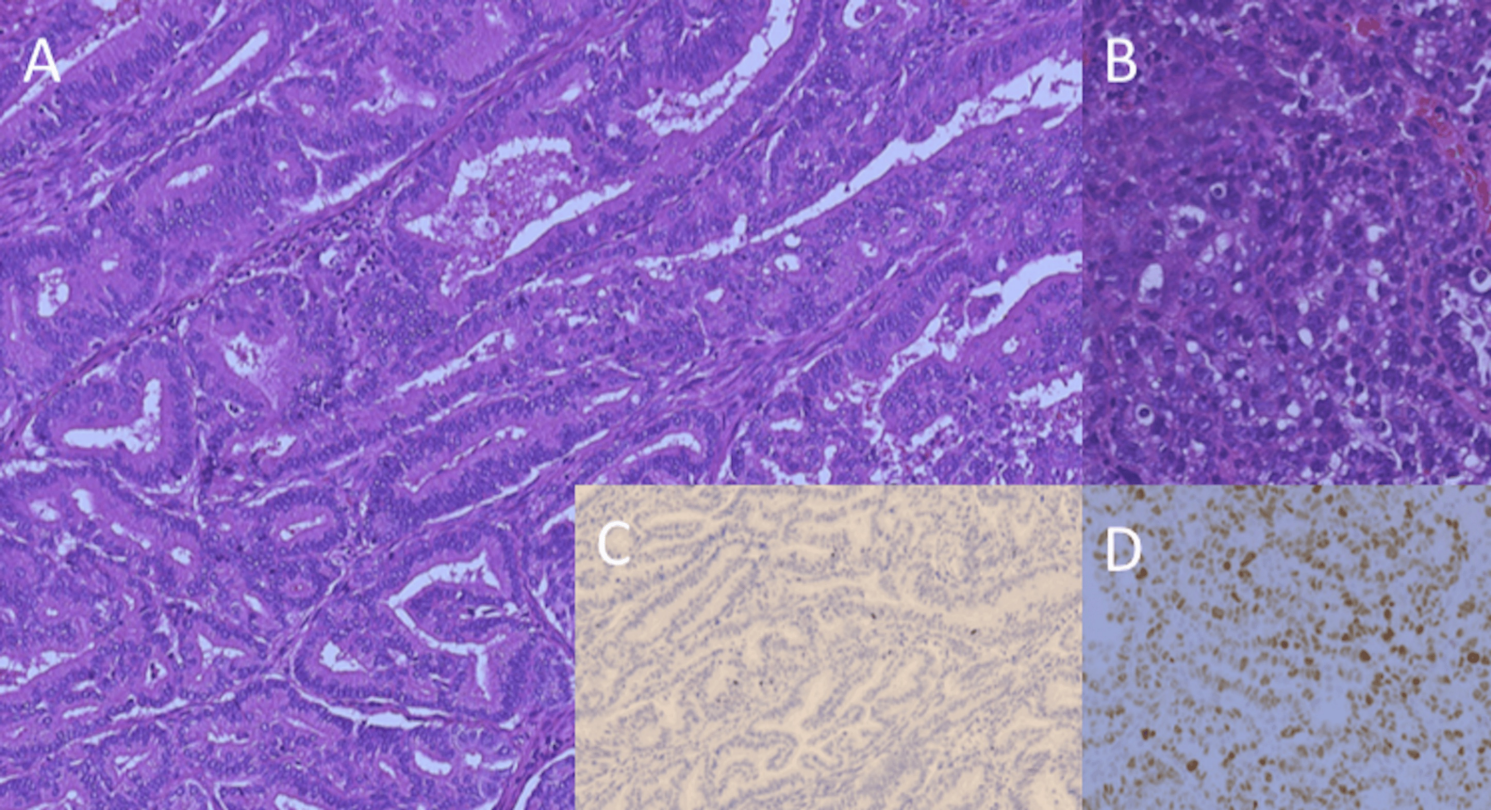
Pathological examination shows a mixed endometrioid (grade 2) (A) and serous carcinoma (B). p53 immunostaining is negative in the glandular component (endometrioid carcinoma) (C) but focally positive in the solid component (serous carcinoma) (D).

In 2012, she underwent surgical staging, consisting of total abdominal hysterectomy, bilateral salpingo-oophorectomy, peritoneal cytology, and pelvic and para-aortic lymphadenectomy. However, a thin capsule (<1 cm in diameter) of a para-aortic lymph node without gross residual disease that was densely adherent to the aortic wall was left in situ, resulting in an R1 resection. Pathological examination revealed a mixed endometrioid (grade 2) and serous carcinoma, and p53 immunostaining was focally positive in the solid component of serous carcinoma (Figure 3). Lymph node metastases were identified in 15 of the 25 nodes removed (7 of 10 para-aortic and 8 of 15 pelvic nodes). The patient was diagnosed with 2008 International Federation of Gynecology and Obstetrics (FIGO) stage IIIC2, pT1B N1 M0 endometrial carcinoma. She received six cycles of adjuvant taxane/platinum-based chemotherapy. After the first cycle of paclitaxel (180 mg/m²)/carboplatin (AUC 5), the regimen was changed to docetaxel (70 mg/m²)/carboplatin (AUC 5) because of neurotoxicity.

**Figure 4:**
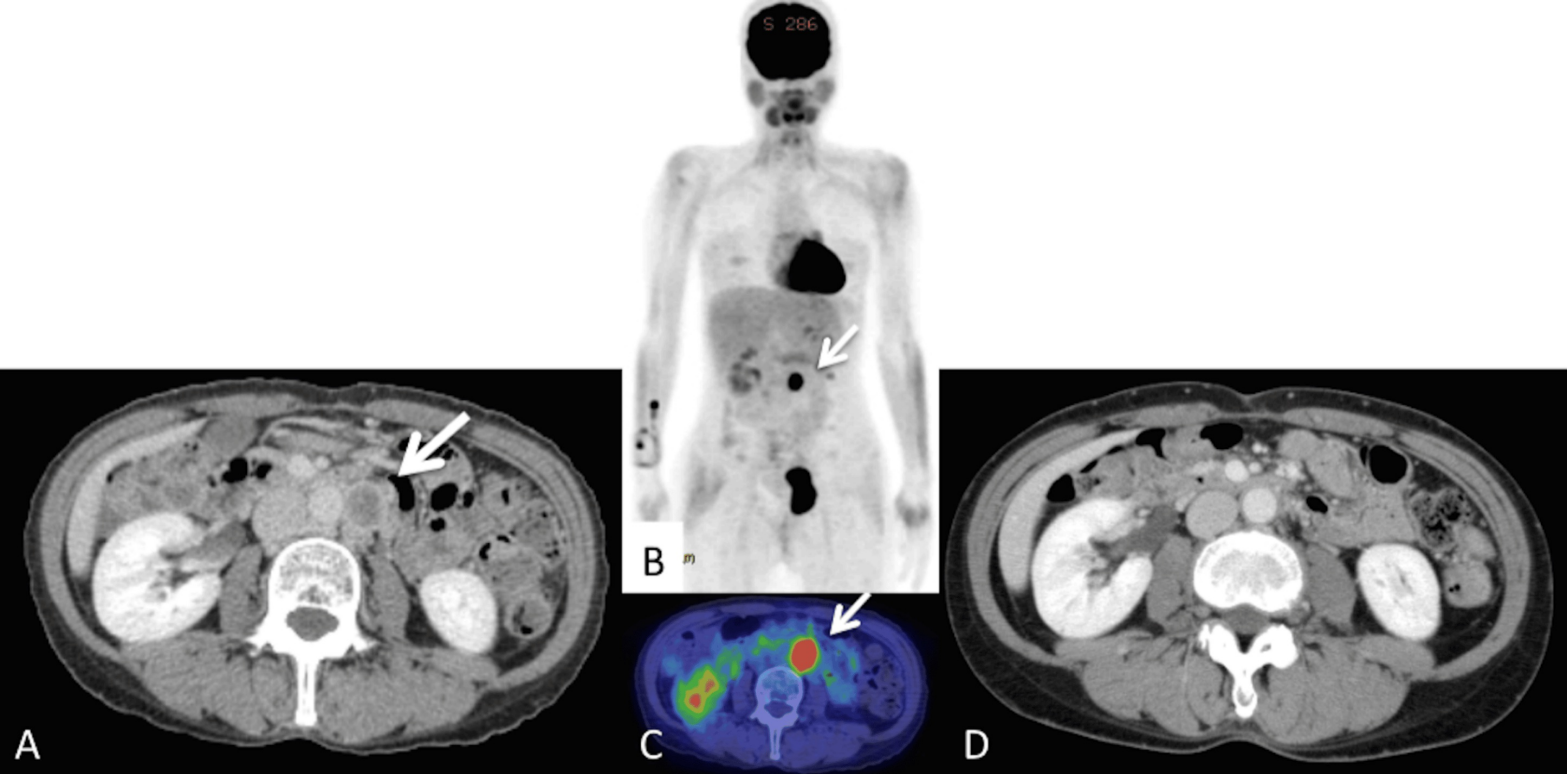
CT scan and PET/CT of a re-enlarged left para-aortic node after surgery and chemotherapy (progressive disease) (A, B, C; arrow). SUVmax of the node was 9.14, and its short axis was 25 mm. After radiotherapy, the enlarged node decreases in size and is considered normal (complete response) (D).

At the completion of chemotherapy, no lymphadenopathy was observed on CT. However, follow-up CT three months later revealed re-enlargement of a para-aortic node, indicating progressive disease (Figures 4A-4C).

No abnormalities were found in the blood test results (Table 1).

**Table 1:** Laboratory data

Variable	At the time of para-aortic recurrence	Reference range
White cell count (10^2^/μL)	53	35-98
Red cell count (10^4^/μL)	428	370-500
Hemoglobin (g/dL)	12.7	11.0-15.3
Hematocrit (%)	39.3	33.0-45.0
Platelet count (10^4^/μL)	23	13.0-37.0
Differential count (%)		
Neutrophils	80	32-79
Eosinophils	0	0-6
Basophils	0	0-2
Monocytes	3	1-8
Lymphocytes	17	18-59
Urea nitrogen (mg/dL)	10	8-22
Creatinine (mg/dL)	0.61	0.6-1.2
Aspartate aminotransferase (U/L)	16	13-33
Alanine aminotransferase (U/L)	13	8-42
Lactate dehydrogenase (U/L)	165	119-229
Alkaline phosphatase (U/L)	138	115-360
Total protein (g/dL)	7	6.7-8.3
Albumin (g/dL)	4.2	3.4-5.8
C-reactive protein (g/dL)	0.04	0.0-0.14
CA125 (U/mL)	19	≤35
CA19-9 (U/mL)	11	0-37

**Figure 5:**
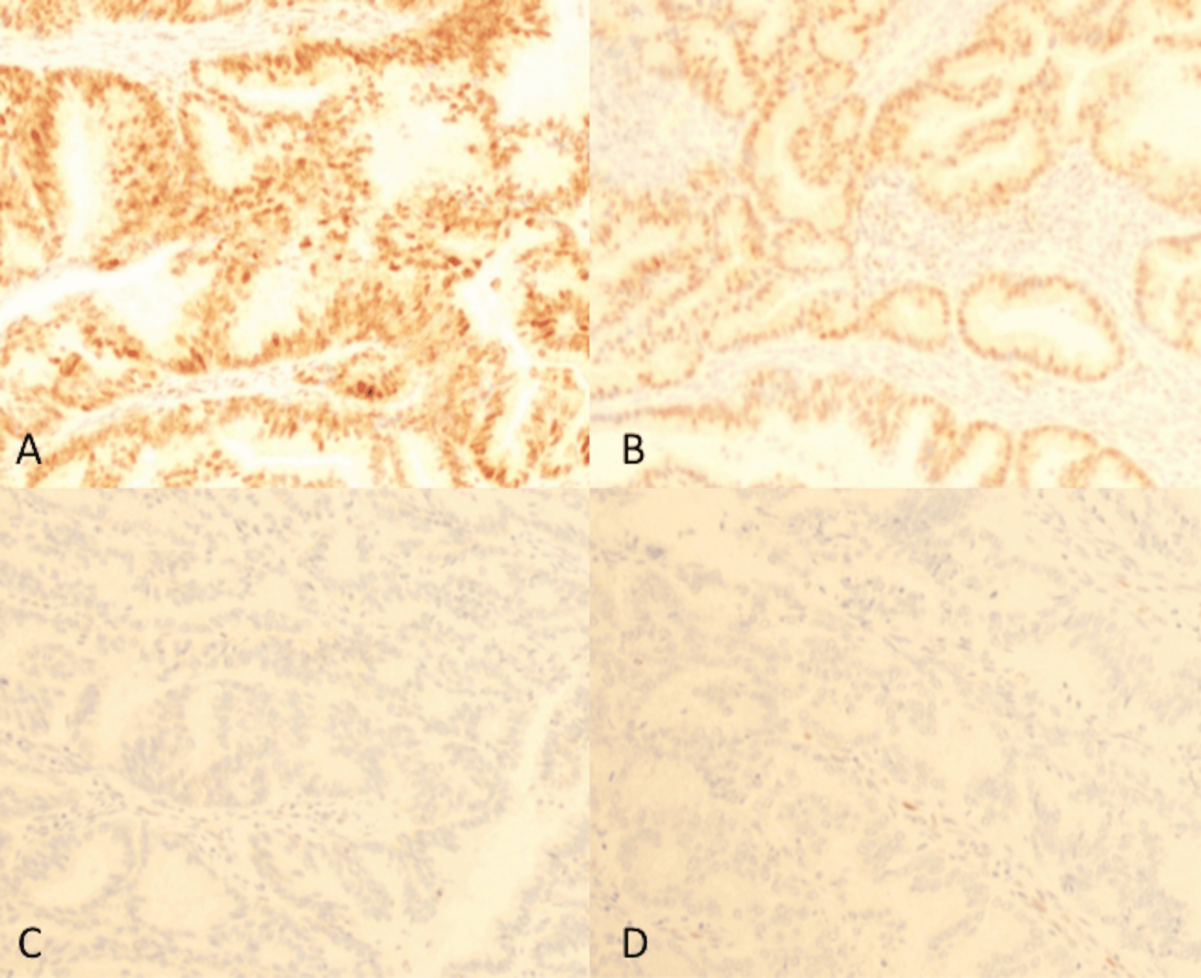
Immunohistochemical staining shows that the tumor cells are positive for MLH1 (A) and PMS2 (B). However, they are negative for MSH2 (C) and MSH6 (D), although the internal positive control was not clearly present.

In 2013, when immune checkpoint inhibitors were not available, external-beam radiotherapy to the para-aortic region was initiated. The clinical target volume (CTV) included the entire para-aortic region extending inferiorly from the L1 vertebra. The planning tumor volume was defined by adding a 1-cm margin around the CTV. The patient was treated with 6 MV X-rays using anterior and posterior parallel opposed fields. After 23.4 Gy in 13 fractions for 19 days, radiotherapy was interrupted for five weeks due to sepsis from an infected pelvic lymphocyst. She ultimately received 59 Gy in 33 fractions for 82 days, achieving a complete response (RECIST 1.1) (Figure 4D). She remains well with no evidence of disease 150 months after surgery. Immunohistochemical staining showed that tumor cells were positive for *MLH1* and *PMS2*. However, they were negative for *MSH2* and *MSH6*, although no clear internal positive control was present on either slide (Figure 5).

## Discussion

This case suggests that radiotherapy may be effective for chemoresistant lymph node metastases from endometrial carcinoma in a woman with suspected Lynch syndrome.

This patient was highly suggestive of Lynch syndrome based on her family cancer history. She fulfilled the Amsterdam II criteria for Lynch syndrome: she was diagnosed at 49 years of age and had a family history of colon cancer in three relatives, including her mother and maternal grandfather. In addition, the tumor histology also supports a possible diagnosis of Lynch syndrome. She had a mixed serous/endometrioid endometrial carcinoma, 33% of which reportedly occur in patients with Lynch syndrome [10]. Endometrial cancer is the most common malignancy in women with Lynch syndrome, and its prevalence among patients with endometrial cancer has been reported to be 5.0% in Japan [11]. Unfortunately, immunohistochemical staining could not definitively confirm MMR deficiency in our case because of the absence of an internal positive control. Nevertheless, the pattern of MMR protein expression -- loss of *MSH2* and *MSH6* with retention of *MLH1* and *PMS2* -- was suggestive of Lynch syndrome associated with an *MSH2* mutation.

Endometrial cancers with MMR deficiency have been observed in approximately 30% of cases, and in these cases, adjuvant chemotherapy may not be effective [2-4]. An intact MMR system is necessary in the response to chemotherapy because it can directly signal cellular apoptotic pathways [12], and cells lacking MMR function fail to receive this signal, thereby preventing apoptosis [2]. In addition, platinum sensitivity has been amplified by restoring MMR in deficient cell lines [13]. A human endometrial cancer cell line deficient in *hMSH2* demonstrated 1.8-fold resistance to cisplatin and 1.5-fold resistance to carboplatin [13].

In contrast, radiotherapy appears to be effective in MMR-deficient endometrial cancers [3,8]. Its clinical efficacy is explained by the induction of lethal DNA damage in tumor cells or the tumor-associated stroma [6]. Additionally, ionizing radiation enhances tumor-specific immune responses [6,14], as it generates large quantities of tumor antigens through necrotic and apoptotic tumor cells and cellular debris. This antigen release stimulates an immune response, thus ionizing radiation may hypothetically convert the irradiated tumor into an efficient individualized in situ vaccine [15]. Localized irradiation increases both the generation of antitumor effector cells and their trafficking to the tumor site [14]. Notably, activated T cells can also eliminate tumor cells at distant sites, known as the abscopal effect.

In a patient with Lynch syndrome, an enhanced radiation-induced tumor-specific immune response would be expected.

In Lynch syndrome-associated cancer cells, insertion and deletion mutations that occur during DNA replication accumulate due to MMR deficiency. These mutations affecting coding microsatellites result in a high burden of frameshift peptide neoantigens. Patients with Lynch syndrome-associated MMR deficiency exhibit a significantly higher burden of somatic mutations and neoantigens compared with patients with sporadic MMR-deficient tumors [16]. Dendritic cells take up tumor neoantigens and then migrate to the lymph node, where they present antigens to T cells. Activated cytotoxic T cells will migrate to the tumor and kill the tumor cells [17]. Tumor-infiltrating lymphocytes are significantly more abundant in Lynch syndrome-associated endometrial cancers than in sporadic cases [16]. However, a harsh microenvironment at the tumor sites may be a physical barrier to T cells. As radiotherapy kills the tumor cells continuously, new antigens are released, and an anti-tumor immune cycle continues [17]. However, these mechanisms remain hypothetical. Multiple factors, including surgical resection of macroscopic disease, systemic inflammation from sepsis, and the prolonged radiotherapy course, may have also contributed to the complete and durable clinical response in our case.

Endometrial cancer with para-aortic node metastases (stage IIIC2) typically requires systemic therapy, as distant metastasis is the most common pattern of failure [18]. However, stage IIIC2 patients with non-endometrioid endometrial cancer had poor survival, even though they received radiotherapy and chemotherapy [18]. In our case, only focal p53 immunostaining in the tumor suggests that the p53 abnormality would presumably be a secondary event following MMR deficiency [19]. This sequence may explain the high radiosensitivity observed, despite the tumor being a mixed serous/endometrioid carcinoma.

Although immune checkpoint inhibitors are effective for MMR-deficient tumors, serious or even life-threatening immune-related adverse events (irAEs) may occur due to indiscriminate activation of T cells. Radiotherapy, which is not associated with irAEs, may be an effective alternative for selected patients. A case of advanced prostate cancer with an abnormal immunohistochemical expression of *MSH2* and *MSH6* that was resistant to chemotherapy and hormone therapy has been reported to show a sustained response to palliative radiotherapy [8]. In a case of MMR-deficient endometrial cancer with loss of *MLH1* and *PMS2* treated with immunotherapy, repeated abscopal effects were observed with radiotherapy [3].

The main limitation of this study is that the diagnosis of Lynch syndrome remains presumptive, as germline confirmation was not performed and immunohistochemistry lacked internal positive controls. The patient declined genetic testing because this high-cost test provides no clinical benefit over regular check-ups for her and her children. The failure of immunohistochemical staining may have resulted from antigen decay, as the staining was performed more than 12 years after the initial resection. Additionally, as this is a single-case study, these results may not be generalizable. Radiotherapy may not always be effective for chemoresistant MMR-deficient tumors. Patients with MutL (*MLH1*/*PMS2*) deficiency may have less immune status and immunotherapy benefits than those with MutS (*MSH2*/*MSH6*) deficiency [20].

## Conclusions

Chemoresistant lesions may respond well to radiotherapy in a patient with suspected Lynch syndrome. Radiotherapy may be a promising option for chemoresistant lesions in selected similar cases. Further studies are warranted to identify the specific subgroup of patients who are likely to benefit from radiotherapy.
